# Supplementary material for: Measuring facial mimicry: Affdex vs. EMG
Source: PLoS One. 2024 Jan 2;19(1):e0290569. doi: 10.1371/journal.pone.0290569 (PMC10760767; doi:10.1371/journal.pone.0290569)
Supplement: Attachment — Submitted filename: Review_PONE-D-22-35210_Init_Submission.pdf [file pone.0290569.s001.pdf]

In the manuscript titled "Measuring facial mimicry: Automated facial coding vs. EMG," Westermann et al. showed participants dynamic facial expressions of various emotions and simultaneously videotaped the participants and recorded the EMG of Zygomaticus major (ZM) and Corrugator supercilii (CS). They correlated EMG activities of ZM and CS with Affdex-estimated values of corresponding action units (AU) over 25 time points of 200 ms intervals. They reported that Affdex could reliably detect spontaneous facial mimicry of smile expressions. However, several factors adversely affected the valid estimation of Affdex's performance detecting spontaneous facial mimicry of frowning expressions.

I agree with the authors that video-based automatic FACS coding tools are very convenient. Several studies have already used such tools for detecting subtle facial movements, such as spontaneous facial mimicry (e.g., Drimalla et al. and Mauersberger et al. [1,2] using OpenFace), without empirical evidence on the validity of automated tools in detecting spontaneous facial mimicry, compared to the golden standard of EMG. This could potentially lead to biased conclusions on the mimicking behavior, particularly when made against a specific population, such as individuals with an autistic diagnosis. An adverse scenario could be that inferences were made that a particular population "lacks" spontaneous facial mimicry only because the measuring methods used (e.g., automated FACS coding) were not sensitive enough. I appreciate the authors' efforts in evaluating such approaches' fundamental issue of measurement validity.

I found issues in study design, statistics, and inference-making problematic. The suitability of publication will be evaluated after a major revision properly addressing the following points.

Major points:

1. Currently, there are several automated FACS coders, including at least two open-source options, OpenFace [1,2] and Py-Feat (https://py-feat.org/), able to integrate with more state-of-the-art deep learning (neural network) models for the same purpose. Under the current situation, it is unclear why the authors chose Affdex, which requires a license, and provides fewer AU estimates, with the only option of the SVM model. The rationale behind the toolbox choice for estimation requires clarification and elaboration.
2. Since the authors only estimated the performance of Affdex, it is to be determined whether their results could be generalized to other automated FACS coding toolboxes trained with different algorithms. Please revise the title to clarify that the present study focused only on Affdex.
3. Line 95: "EMG is relatively invasive due to the use of measuring electrodes on the face and, a bias of the results is conceivable." This sentence requires elaboration and supporting empirical evidence. Facial EMG using surface electrodes (just like EEG) did not necessarily irritate 4-month-old infants [3], and the so-called "bias of the results" was

vague and unspecified.

4. Line 216: Please specify "the clinically defined thresholds" for subject exclusion to make this study reproducible. Also, in Table 1, please include the range of trait measurements.
5. In FACS, AUs were clearly numbered and named. The AUs that corresponded to ZM and CS were AU12, "Lip corner puller," and AU4, "Brow lowerer," which were also listed so in the publication of Affdex [4]. In the present manuscript, the authors used the term "smile action unit," which was confusing and possibly misleading. The smiling action involves at least AU12 and AU6 (Cheek raiser) [5]. Did the authors use the average of multiple AU measures (AU12 and 6) to correlate with ZM activity or only AU12? Please clarify and follow the proper FACS terms.
6. Statistics:
    a. For each emotion, participants were tested eight times (two age groups, two genders, and two runs). In the EMG data, separate left and right face measurements were averaged during the integration. In the Affdex data, data from stimuli of two genders were averaged. How did the authors deal with repeated measures of two age groups, runs, and genders in the EMG and Affdex data? Furthermore, the authors could use algorithms incorporating mixed effects to take advantage of the statistical power of repeated measures.
    b. The authors performed 25 spearman correlations along the time series for each ZM and CS data. First, this approach treated time points as independent and identically distributed (IID), which might be used to infer that "when the measurement of CS is high, the measurement of AU4 by Affdex would be high". This approach ignored the fact that both measurements were time series with characteristics of autocorrelation, trends, seasonality, etc. Second, the issue of alpha correction for multiple testing was not addressed. I suggest the use of algorithms testing whether "two time-series were correlated/showing the same trend," which also considers the repeated measure design, such as the "Repeated measures correlation" (rmcorr R package), testing whether ZM correlated with AU12, and CS correlated with AU4, using "subject" as a random factor, include all repeated measures without averaging, and use the option "bootstrapping" for confidence interval estimation.
    c. Since there was a noticeable time lag between the muscle activity (EMG) and the facial landmark changes (AU) with reasonable explanations, the authors should consider testing cross-correlations between EMG and AU time series. The authors should consider other statistical algorithms appropriate for testing their hypothesis.
    d. Reports of statistical results should include test statistics (r), degrees of freedom, effect sizes, confidence intervals, and exact corrected p-values, as per PLOS One statistical guideline (https://journals.plos.org/plosone/s/submission-

guidelines.#loc-statistical-reporting).

7. It is unclear how Affdex was trained for emotion expression classification. McDuff et al. [4] only described that “The emotion expressions are based on combinations of facial actions. This coding was built on the emotional facial action coding system”. Was the SVM algorithm also used for emotion expression classification based on the AU patterns?
8. Sato and Yoshikawa [6] reported latency of maximally 900 ms post-stimulus onset when using automated FACS, while the present study reported a 1200 ms latency for smile mimicry. Could this indicate a lower sensitivity of Affdex compared to other automated FACS?
9. There were several reasons why the angry mimicry was not detected by Affdex, one of which concerns that the placement of CS electrodes adversely affected the Affdex estimation. It is still being determined whether retouching the video data for electrode placements is valid data processing. In this case, the null results did not exemplify that Affdex “cannot detect” angry mimicry. Instead, in the study setup, the placement of electrodes confounded Affdex’s ability to estimate AU4. I suggest modifying the inference and claiming that the present study is not suitable for estimating Affdex’s performance in AU4, as discussed in the second half of the Discussion. I also agree with the authors that the initial validity evaluation of automated FACS should start with comparing the performance of human FACS coders and automated FACS coders.
10. While the data reporting included the data tables, the code for statistical analysis needed to be included; please also include the code for the study reproducibility.

Minor points:

11. After formatting the citation style, the authors did not check the manuscript's readability. In numerous instances, such as in line 72, “18 (18) showed that the presentation of happy faces led to an increase in EMG activity…” the sentence does not make sense. The first “18” should have been the author of reference No. 18, Dimberg, while “(18)” referred to reference 18. I suggest the authors check the manuscript thoroughly before resubmission.
12. Section 3.1: abbreviations of interviews and questionnaires were mentioned before the full terms were introduced. When introducing the full terms, such as the “Structured Clinical Interview for DSM-IV” in line 160, the abbreviations did not follow the full term. This also negatively affected readability.
13. Lines 492 & 499: The same sentence was repeated twice – “35 (35) studied a healthy cohort who imitated faces with maximum affect expression. Here, Affdex measured a maximum mean of….” I suggest the authors reinspect and reorganize this paragraph. See also point 11.

Bibliography

1. Baltrušaitis T, Mahmoud M, Robinson P. Cross-dataset learning and person-specific normalisation for automatic Action Unit detection. 2015 11th IEEE International Conference and Workshops on Automatic Face and Gesture Recognition (FG). 2015. pp. 1–6. doi:10.1109/FG.2015.7284869

2. Baltrušaitis T, Zadeh A, Lim YC, Morency L-P. OpenFace 2.0: Facial Behavior Analysis Toolkit. 2018 13th IEEE International Conference on Automatic Face & Gesture Recognition (FG 2018). 2018. pp. 59–66. doi:10.1109/FG.2018.00019

3. de Klerk CCJM, Hamilton AF de C, Southgate V. Eye contact modulates facial mimicry in 4-month-old infants: An EMG and fNIRS study. Cortex. 2018;106: 93–103. doi:10.1016/j.cortex.2018.05.002

4. McDuff D, Mahmoud A, Mavadati M, Amr M, Turcot J, Kaliouby R el. AFFDEX SDK: A Cross-Platform Real-Time Multi-Face Expression Recognition Toolkit. Proceedings of the 2016 CHI Conference Extended Abstracts on Human Factors in Computing Systems. San Jose California USA: ACM; 2016. pp. 3723–3726. doi:10.1145/2851581.2890247

5. Cordaro DT, Sun R, Keltner D, Kamble S, Huddar N, McNeil G. Universals and cultural variations in 22 emotional expressions across five cultures. Emotion. 2018;18: 75–93. doi:10.1037/emo0000302

6. Sato W, Yoshikawa S. Spontaneous facial mimicry in response to dynamic facial expressions. Cognition. 2007;104: 1–18. doi:10.1016/j.cognition.2006.05.001
