# Supplementary material for: Measuring facial mimicry: Affdex vs. EMG
Source: PLoS One. 2024 Jan 2;19(1):e0290569. doi: 10.1371/journal.pone.0290569 (PMC10760767; doi:10.1371/journal.pone.0290569)
Supplement: Attachment — Submitted filename: Review_PONE-D-22-35210_R1.pdf [file pone.0290569.s003.pdf]

The authors have addressed the reviewers' comments adequately.

There are three points left:

1. Fig. 1 to 4: The asterisk * highlighting significant Spearman correlations should be based on corrected p-values. Currently, the figures still highlight uncorrected p-values, so when there is no significant correlation after alpha correction for multiple comparisons in Table 5, there are still two asterisks in Fig. 4. The discrepancy between corrected p-values and asterisks also applies to Fig. 1 and 3.
2. Line 569-570: the problematic description "In addition, EMG is relatively invasive due to the use of measuring electrodes on the face, and bias in the results is conceivable" remained. Please revise it to make it consistent with the revised information in the Introduction.
3. After seeing the result of the repeated measures correlations and cross-correlations, I found that the cross-correlations provided statistical evidence about a lagged matching in temporal patterns between EMG and Affdex (augmenting the serial Spearman correlations in Fig. 1 to 4, from which such an inference could only be made with visual inspection and comparing the peaking time of two time-series). In contrast, repeated measures correlations collapsing all time points eventually said little beyond the relatively unspecific fact that the time series are correlated somehow. Ultimately, I leave it to the authors to decide whether they will include the repeated measures correlations, cross-correlations, or not at all.
