# Supplementary material for: Measuring facial mimicry: Affdex vs. EMG
Source: PLoS One. 2024 Jan 2;19(1):e0290569. doi: 10.1371/journal.pone.0290569 (PMC10760767; doi:10.1371/journal.pone.0290569)
Supplement: Attachment — Submitted filename: Response to Reviewers.docx [file pone.0290569.s004.docx]

## Klinisches Institut für Psychosomatische Medizin und Psychotherapie, Geb. 15.16

# Komm. Direktor

**Univ.-Prof. Dr. med. M. Franz**

Tel (0211) 81-18338

[matthias.franz@uni-duesseldorf.de](mailto:matthias.franz@uni-duesseldorf.de)

**Vorzimmer**

**D. Leth**

Tel (0211) 81-18338 (Fax -16250)

[dagmar.leth@med.uni-duesseldorf.de](mailto:dirk.rampoldt@med.uni-duesseldorf.de)

**Oberarzt/stellv. Institutsleiter**

**Dr. med. A. Karger, MME**

Tel (0211) 81-16212

[andre.karger@med.uni-duesseldorf.de](mailto:andre.karger@med.uni-duesseldorf.de)

**Oberärztin**

**Dr. med. F. Sareika**

Tel (0211) 81-08117

[frauke.sareika@med.uni-duesseldorf.de](mailto:frauke.sareika@med.uni-duesseldorf.de)

**Sekretariat Lehre und Forschung**

**C.-E. Schikorra**

Tel (0211) 81-08061 (Fax -16250)

[cora-ellen.schikorra@med.uni-duesseldorf.de](mailto:cora-ellen.schikorra@med.uni-duesseldorf.de)

**Funktionsbereiche**

- **Allg. Konsil- und Liaisondienst**
- **Psychoonkologie**
- **Psychokardiochirurgie/-kardiologie**
- **Psychodiabetologie**
- **UKD Mitarbeiterberatung**

**Patientenanmeldung**

**A. Arnold**

Tel (0211) 81-18855 (Fax -16250)

[anja.arnold@med.uni-duesseldorf.de](mailto:anja.arnold@med.uni-duesseldorf.de)

**Psychosomatischer Konsildienst**

**K. Franzmeyer, Ärztin**

Tel (0211) 81-08235

[karin.franzmeyer@med.uni-duesseldorf.de](mailto:karin.franzmeyer@med.uni-duesseldorf.de)

**Dr. med. L. S. Fröhlich, Ärztin**

Tel (0211) 81-18304

[lindasophie.froehlich@med.uni-duesseldorf.de](mailto:lindasophie.froehlich@med.uni-duesseldorf.de)

**Dr. med. D. Fugmann, Arzt**

Tel (0211) 81-17354

[dominik.fugmann@med.uni-duesseldorf.de](mailto:dominik.fugmann@med.uni-duesseldorf.de)

**C. Lange, Ärztin**

Tel (0211) 81-08042

[christinaregina.lange@med.uni-duesseldorf.de](mailto:christinaregina.lange@med.uni-duesseldorf.de)

**J. Ramm, Ärztin**

Tel (0211) 81-18855

[jaqueline.ramm@med.uni-duesseldorf.de](mailto:jaqueline.ramm@med.uni-duesseldorf.de)

**K. Sättler, M.Sc. Psychologin**

Tel (0211) 81-08236

[kristina.saettler@med.uni-duesseldorf.de](mailto:kristina.saettler@med.uni-duesseldorf.de)

**Dr. med. F. Schiffers, Ärztin**

Tel (0211) 81-04081

[freya.schiffers@med.uni-duesseldorf.de](mailto:freya.schiffers@med.uni-duesseldorf.de)

**S. Schipper-Kochems, Ärztin**

Tel (0211) 81-16172

[stephanie.schipper-kochems@med.uni-duesseldorf.de](mailto:stephanie.schipper-kochems@med.uni-duesseldorf.de)

**S. Tießen, M.Sc. Psychologin**

Tel (0211) 81-07795

[SwantjeBritteDorothea.Tiessen@med.uni-duesseldorf.de](mailto:SwantjeBritteDorothea.Tiessen@med.uni-duesseldorf.de)

**Dr. med. J. von Schreitter, Ärztin**

Tel (0211) 81-08235

[julia.vonschreitter@med.uni-duesseldorf.de](mailto:julia.vonschreitter@med.uni-duesseldorf.de)

**J. Westermann, Arzt**

Tel (0211) 81-17354

[JanFrederik.Westermann@med.uni-duesseldorf.de](mailto:JanFrederik.Westermann@med.uni-duesseldorf.de)

**Integrierte Psychoonkologische**

**Versorgung (AOK), Lotsin**

**D.M. Pukat, M.Sc. Psychologin**

Tel (0211) 81-18188

[lotse@med.uni-duesseldorf.de](mailto:lotse@med.uni-duesseldorf.de)

**Psychosoziale Beratungsstelle**

**J. Riemer, M.A. Coaching/Supervision**

Tel (0211) 81-16160 (Fax -19760)

[riemer@med.uni-duesseldorf.de](mailto:riemer@med.uni-duesseldorf.de)

Psychophysiologisches Labor

**Dr. phil. Dipl.-Psych. R. Schäfer**

**stellv. Laborleiter/ltd. Psychologe**

Tel (0211) 81-12536 Labor, 81-18308 Büro

[schaefra@uni-duesseldorf.de](mailto:schaefra@uni-duesseldorf.de)

**M.A. Nordmann, Diplom-Psychologe**

Tel (0211) 81-12536

[marc.nordmann@med.uni-duesseldorf.de](mailto:marc.nordmann@med.uni-duesseldorf.de)

| Universitätsklinikum Düsseldorf AöR • Moorenstraße 5 • 40225 Düsseldorf |  |
| --- | --- |

| Phone: | E-Mail: | Date: |
| --- | --- | --- |
| +49 163 494 0710 | westermannjan@yahoo.de | 26.07.2023 |
|  |  |  |

**Measuring facial mimicry: Affdex vs. EMG**

**(Former title: Measuring facial mimicry: Automated facial coding vs. EMG)**

Dear Editors,

We would like to thank you for your letter, the helpful comments and the opportunity to resubmit a revised copy of our manuscript.

Your constructive feedback helped us to improve our paper and to focus on the most important results of our study. In the following, we will respond to each point raised by the reviewers and will refer to the changed text passage.

**Reviewer #1.**

1. Fig. 1 to 4: The asterisk * highlighting significant Spearman correlations should be based on corrected p-values. Currently, the figures still highlight uncorrected p-values, so when there is no significant correlation after alpha correction for multiple comparisons in Table 5, there are still two asterisks in Fig. 4. The discrepancy between corrected p-values and asterisks also applies to Fig. 1 and 3.

We checked the figures and marked only those correlation coefficients that were significant after alpha correction for multiple comparisons.

2. Line 569-570: the problematic description “In addition, EMG is relatively invasive due to the use of measuring electrodes on the face, and bias in the results is conceivable” remained. Please revise it to make it consistent with the revised information in the Introduction.

We have removed the problematic description: “In addition, EMG is relatively invasive due to the use of measuring electrodes and a bias is conceivable.”

3. After seeing the result of the repeated measures correlations and cross-correlations, I found that the cross-correlations provided statistical evidence about a lagged matching in temporal patterns between EMG and Affdex (augmenting the serial Spearman correlations in Fig. 1 to 4, from which such an inference could only be made with visual inspection and comparing the peaking time of two time-series). In contrast, repeated measures correlations collapsing all time points eventually said little beyond the relatively unspecific fact that the time series are correlated somehow. Ultimately, I leave it to the authors to decide whether they will include the repeated measures correlations, cross-correlations, or not at all.

We agree with the statement that cross correlations provide evidence for lagged matching in temporal patterns between EMG and Affdex. In addition, Reviewer#2 in Point 5 also recommends, among other things, performing cross correlation calculations. Therefore we decided to include cross-correlations.

**Reviewer#2.**

1. 1The paragraph starting line 50 does not accurately present the current state of knowledge on the relationship between facial mimicry and facial emotion recognition. The results are contentious and should be presented as such. I urge the authors to tone down claims and statements that facial mimicry is involved in facial emotion recognition and present the evidence for a link as well as counter-evidence (e.g., https://pubmed.ncbi.nlm.nih.gov/11165351/).

further caution the authors in using the term 'blocked' when describing facial muscle manipulations. The authors of the cited studies induced facial muscle activity that interfered with mimicry. The terminology of 'blocking' would be more appropriate for Botox studies.

We have adjusted the section according to your suggestions. We think your suggestions is important and enriches the introduction.

2. is unclear what is meant by this sentence: "Similarly, an increase in mimic muscle activity due to, e.g., a task, may lead to lower accuracy in recognizing facial expressions". Please rephrase.

We have extended the sentence as follows: “Similarly, an increase in mimic muscle activity (e.g., due to a task such as biting on a pen or holding a pen with the lips), may result in lower accuracy in facial expression recognition.”

3. The study cited in line 96 [32] is incorrectly presented, as the results did not show group differences in the facial mimicry response.

We have removed the citation.

4. line 102: Using abrasive gel is no necessity. I have never used abrasives on participant's faces, as the cleaning with an alcohol swap is sufficient for good data quality. This procedure is also more pleasant for participants.

We have removed the statement and are considering cleaning the skin of the test persons with an alcohol swap only.

5. In terms of the analysis approach, I suggest the authors follow one of the methods presented here: https://towardsdatascience.com/four-ways-to-quantify-synchrony-between-time-series-data-b99136c4a9c9

Given that the data presented in the manuscript constitutes a time series, it would be appropriate and more parsimonious to conduct time series analyses only.

We chose our approach because it allows us to see over time at what point the EMG measurement and the Affdex measurement correlate significantly with each other. It is true that this method is not optimally suited to correlate time series with each other. Therefore, we calculated lagged cross correlations as presented on the internet page you suggested and as suggested by Reviewer #1. We integrated the results into the manuscript. We have mapped all four plots here in the rebuttal letter. We decided to show only the first plot as an example in the manuscript and to add the other results in a table in results section.

6. line 550: I suggest to delete the part 'relatively invasive'. In addition, participants generally report getting used to the electrodes within minutes and not even 'feeling' them anymore over the course of the experiment.

We have removed the problematic description: “In addition, EMG is relatively invasive due to the use of measuring electrodes and a bias is conceivable.”

**Changes to the reference list:**

Removed references:

1. Bakdash JZ, Marusich LR. Corrigendum: Repeated Measures Correlation. Frontiers in psychology 2019;10:1201. PubMed PMID: 31191398; PubMed Central PMCID: PMC6548204.

Reason: Repeated Measures Correlations were not conducted in the current version.

2. Passardi S, Peyk P, Rufer M, Wingenbach TSH, Pfaltz MC. Facial mimicry, facial emotion recognition and alexithymia in post-traumatic stress disorder. Behav Res Ther 2019;122:103436. PubMed PMID: 31557692.

Reason: The study was presented incorrectly.

3. Kulke L, Feyerabend D, Schacht A. A Comparison of the Affectiva iMotions Facial Expression Analysis Software With EMG for Identifying Facial Expressions of Emotion. Frontiers in psychology 2020;11:329. PubMed PMID: 32184749; PubMed Central PMCID: PMC7058682.

Reason: The reference was in reference list twice.

New references:

1. Niedenthal PM, Barsalou LW, Winkielman P, Krauth-Gruber S, Ric F. Embodiment in attitudes, social perception, and emotion. Pers Soc Psychol Rev 2005;9(3):184–211. PubMed PMID: 16083360.

Reason: Extension of the introduction.

2. Niedenthal PM, Wood A, Rychlowska M, Korb S, editors. Embodied Simulation in Decoding Facial Expression:In J.-M. Fernández-Dols & J. A. Russel (Eds.), The Science of facial expression (pp. 397-414).: Oxford University Press; 2017. 25 p.

Reason: Extension of the introduction.

3. Gump BB, Kulik JA. Stress, Affiliation, and Emotional Contagion. Journal of Personality and Social Psychology 1997;72(2):305–19.

Reason: Extension of the introduction.

4. Seibt B, Weyers P, Likowski KU, Pauli P, Mühlberger A, Hess U. Subliminal Interdependence Priming Modulates Congruent and Incongruent Facial Reactions to Emotional Displays. Social Cognition 2013;31(5):613–31.

Reason: Extension of the introduction.

5. Lanzetta JT, Englis BG. Expectations of cooperation and competition and their effects on observers' vicarious emotional responses. Journal of Personality and Social Psychology 1989;56(4):543–54.

Reason: Extension of the introduction.

We are very glad about your helpful and high quality comments. We are sure that your contributions have greatly enriched our work and we hope that you agree with our implementation. We are already looking forward to your next feedback!

Please address all correspondence concerning this manuscript to westermannjan@yahoo.de.

Thank you for taking this manuscript into consideration.

Cordially

Prof. Dr. M. Franz
